# Overestimation of volatility in schizophrenia and autism? A comparative study using a probabilistic reasoning task

**DOI:** 10.1371/journal.pone.0244975

**Published:** 2021-01-07

**Authors:** Isabel Kreis, Robert Biegler, Håkon Tjelmeland, Matthias Mittner, Solveig Klæbo Reitan, Gerit Pfuhl

**Affiliations:** 1 Department of Psychology, Faculty of Health Sciences, UiT–The Arctic University of Norway, Tromsø, Norway; 2 Department of Psychology, Faculty of Social and Educational Sciences, Norwegian University of Science and Technology, Trondheim, Norway; 3 Department of Mathematical Sciences, Faculty of Information Technology and Electrical Engineering, Norwegian University of Science and Technology, Trondheim, Norway; 4 Department of Mental Health, Faculty of Medicine and Health Sciences, Norwegian University of Science and Technology, Trondheim, Norway; 5 Department of Mental Health, St Olav’s University Hospital, Trondheim, Norway; Dartmouth College, UNITED STATES

## Abstract

**Background and objectives:**

A plethora of studies has investigated and compared social cognition in autism and schizophrenia ever since both conditions were first described in conjunction more than a century ago. Recent computational theories have proposed similar mechanistic explanations for various symptoms beyond social cognition. They are grounded in the idea of a general misestimation of uncertainty but so far, almost no studies have directly compared both conditions regarding uncertainty processing. The current study aimed to do so with a particular focus on estimation of volatility, i.e. the probability for the environment to change.

**Methods:**

A probabilistic decision-making task and a visual working (meta-)memory task were administered to a sample of 86 participants (19 with a diagnosis of high-functioning autism, 21 with a diagnosis of schizophrenia, and 46 neurotypically developing individuals).

**Results:**

While persons with schizophrenia showed lower visual working memory accuracy than neurotypical individuals, no significant group differences were found for metamemory or any of the probabilistic decision-making task variables. Nevertheless, exploratory analyses suggest that there may be an overestimation of volatility in subgroups of participants with autism and schizophrenia. Correlations revealed relationships between different variables reflecting (mis)estimation of uncertainty, visual working memory accuracy and metamemory.

**Limitations:**

Limitations include the comparably small sample sizes of the autism and the schizophrenia group as well as the lack of cognitive ability and clinical symptom measures.

**Conclusions:**

The results of the current study provide partial support for the notion of a general uncertainty misestimation account of autism and schizophrenia.

## Introduction

More than a century ago, the terms ‘autistic’ and ‘autism’ were coined to describe the social withdrawal observed in individuals with schizophrenia (SCZ) and a childhood form of SCZ, respectively [1]. While SCZ and autism spectrum disorders (ASD) are defined as distinct entities today [2], a substantial amount of research has investigated the shared characteristics of both conditions. Findings suggest an association from both a genetic [3, 4] and a cognitive-behavioral perspective, particularly within the social domain. Comparative and parallel studies have documented similarly impaired social cognitive abilities in SCZ and ASD relative to neurotypically developing (NT) individuals [5]. This concerns various subdomains, including theory of mind, i.e. the ability to infer others’ mental states [6, 7], eye gaze on faces [8], trustworthiness judgements and emotion identification [9]. In fact, a recent systematic review concluded that apart from emotion recognition there seem to be no clear and consistent differences between ASD and SCZ in terms of social cognitive performance [10].

While social cognition has been studied extensively, only few studies compare the two conditions in other cognitive domains [1]. However, results of separately conducted studies suggest similar decision-making impairments in non-social situations [11–13]. One decision-making bias that has extensively been investigated in persons with SCZ is the so-called “Jumping-to-Conclusions” (JTC) bias. It is usually assessed by the beads task in which beads are sampled from one out of two possible containers (e.g. bags) containing unlike amounts of differently colored beads. Based on the sampled beads, participants have to indicate what they believe to be the bag of origin [14]. Different versions of the task exist: in draws-to-decision versions, participants are free to sample as many beads as they want until they decide on the bag of origin. Here, the JTC bias is characterized by premature decisions, i.e. a decision on the bag of origin after very few beads have been sampled. In graded estimates versions of the task participants indicate their certainty about the bag of origin after each bead. Here, reasoning biases include high (initial) certainty and over-adjustment of the reported estimates, meaning radical belief alterations in response to objectively only modest disconfirmatory evidence [15, 16]. Those biases are similar to the ‘classical’ JTC bias in that they all concern drastic decision-making in light of little evidence. While typically studied in SCZ, the JTC bias has also been found in ASD [17]. Conversely, Brosnan and colleagues reported that persons with ASD gathered more beads before making a decision [18].

Of the few studies directly comparing non-social decision-making in ASD and SCZ, Zhang and colleagues [19] found similar impairments in decision-making under different kinds of uncertainty, suggesting that both conditions may be characterized by misestimation of uncertainties. Such misestimations could also explain the aberrant behavior observed in the aforementioned beads task, where performance relies on Bayesian inference [20]. This perspective fits well with computational theories proposing similar mechanistic explanations based on misestimation of uncertainty for various symptoms of ASD and SCZ [21–23]. According to these theories, symptoms might be the result of (implicit) uncertainty misestimation on different levels in Bayesian belief hierarchies of the brain [24]. One ‘level’ concerns beliefs about the environment’s volatility, i.e. the probability for the environment to change. The results of various studies indicate that both persons with ASD and persons with SCZ overestimate volatility, i.e. they seem to perceive the world as less stable. For example, they exhibit more (maladaptive) switching behavior in reversal learning tasks than NT individuals (ASD: [12, 25–27]; SCZ: [28–31]). Surprising events are thus attributed to a change in the overall stochastic structure of the environment [32] rather than to known uncertainties on lower levels, i.e. the expected uncertainty that arises naturally since some events are more likely than others in a stable but stochastic environment. Hence, new events will become more salient as they might signal a relevant change in the environment when subjective volatility is high. Consequentially, beliefs are updated more drastically. This fits well with the over-adjustment of beliefs observed in the beads task, which in turn has been attributed to a “hypersalience” of new evidence [20].

Aberrant representation of uncertainties has also been described as a ‘failure of metacognition’ [24]. Metacognition can refer to both conscious reflective thought processes and automatic monitoring of one’s own thoughts and cognitions [33]. Metacognitive performance is often determined by comparing self-reports and confidence ratings to actual performance [34]. Interestingly, impaired metacognition has been found in both SCZ [35, 36] and ASD [37, 38]. Further, previous studies have revealed a relationship between the JTC bias, corresponding decision confidence and metacognitive deficits in SCZ [36, 39, 40], but to what extent metacognition relates to higher level uncertainty estimation such as volatility remains to be elucidated.

A general misestimation of uncertainties could thus explain various cognitive-behavioral findings in both ASD and SCZ but it remains unclear if and to what extent both groups differ from each other when compared directly. This study aimed to investigate this question with a focus on volatility processing in a modified beads task and its relationship to belief updating, metacognition and working memory, to account for the potential role of general cognitive capacity.

## Materials and methods

Persons with SCZ were contacted through a clinician at St. Olavs Hospital, Trondheim University Hospital, Norway, while persons with ASD were recruited through the patient interest group *Autismeforeningen* and, like NT control participants, through fliers and social media posts. Participants had to meet the following inclusion criteria: (1) 18 to 60 years of age, (2) no current suicide intent, (3) no substance dependence, (4) IQ above 80, (5) a primary diagnosis from the schizophrenia spectrum (SCZ group) or high-functioning autism/Asperger (ASD group) or no psychiatric diagnosis at all (NT group). All participants in the SCZ group were inpatients who had previously been diagnosed according to the ICD-10 research criteria [41] in a consensus meeting assessing clinical reports with at least two senior psychiatrists or psychologists present, of which at least one had personally examined the patient. Diagnoses were confirmed by clinicians upon inclusion in the study. All participants in the ASD group reported prior diagnoses by independent clinicians. Where available, their diagnoses were confirmed through clinical records and their employer (a business exclusively employing persons with a confirmed ASD diagnosis). For three participants with ASD, no such confirmation was available. For all participants, written informed consent was obtained prior to the study. The study was approved by the Central-Norwegian regional committee for medical and health research ethics (REC Central; reference no.: 2014/1648). In total, 92 participants were recruited, whereof six were excluded since they did not complete enough (≥ 80%) trials of the administered tasks. A subset of the participants filled out additional questionnaires but those results are not reported here.

### Measures

#### Beads task

**Fig 1:**
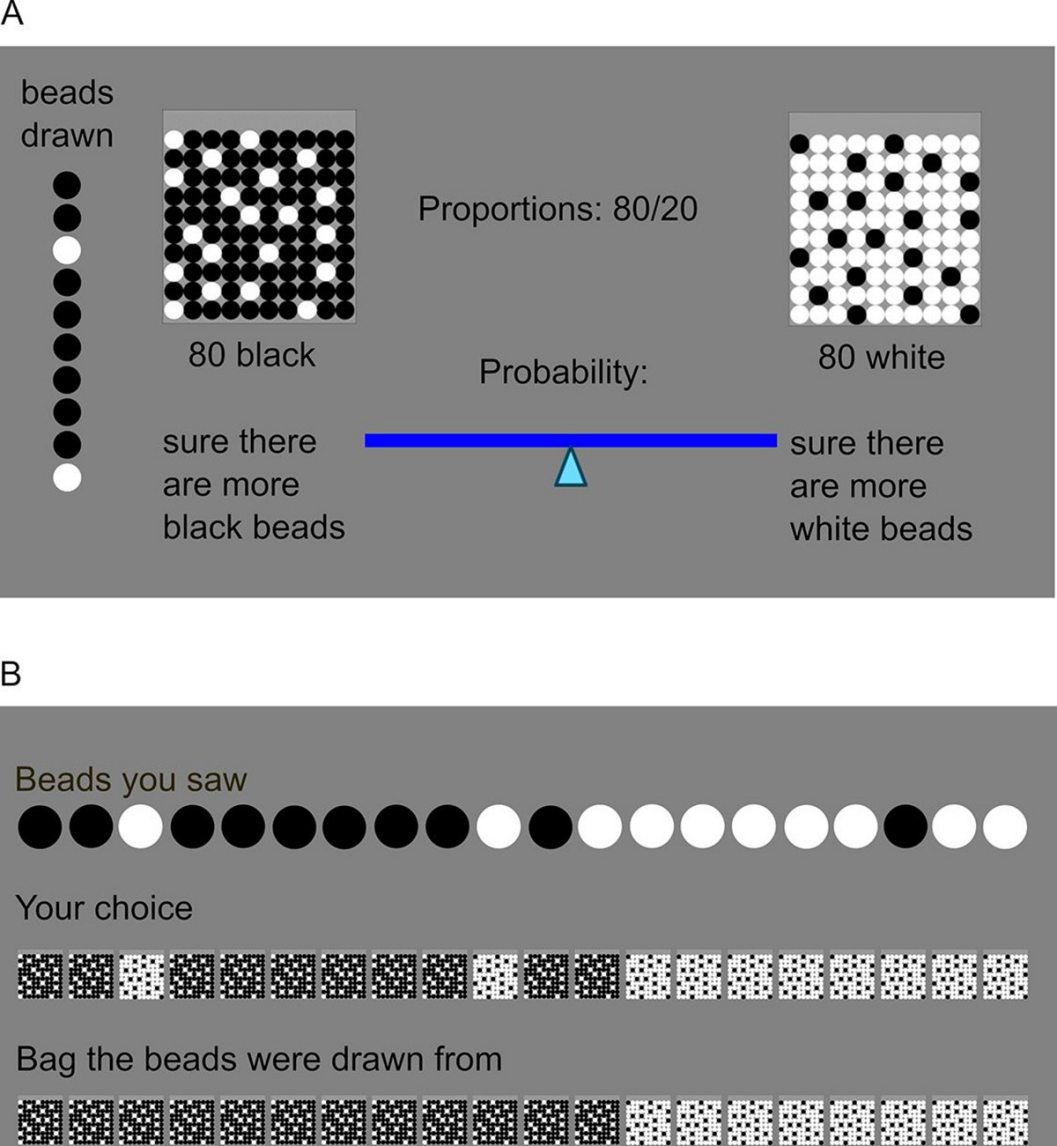
Schematic representation of the beads task. (A) Example of the 10th trial of one sequence. Two bags are displayed which contain either 80 black and 20 white beads, or the converse. Beads are drawn sequentially with replacement. Each of the five sequences consists of 20 drawn beads and the result of each draw, i.e. the color of the bead, remains displayed on the left side of the screen. Within 10 seconds, participants have to indicate their certainty about the bag of origin. They do so by dragging the marker on a visual scale either to the left or the right side. This slider is reset to the center after every trial. (B) At the end of each sequence, feedback about beads seen, choices made and the actual bag of origin of all beads is provided.

To measure probabilistic decision-making and subjective volatility, a modified version of the beads task was administered (see Fig 1). Two virtual bags were displayed on screen, containing 80 black and 20 white beads and the converse. Five sequences of 20 beads each were presented to the participants. At the beginning of each sequence, one of the two bags was chosen at random (*p* = 0.5). Each sequence was then generated based on the probabilities for the different colors to be drawn (*p* = 0.2 and *p* = 0.8) and a fixed probability for the bag of origin to change (*v* = 0.04 for each bead, amounting to a ca. 50% chance to observe a bag change in one sequence). This change probability introduced volatility to the task. Participants were informed about this by written instructions stating: “The chance for the bags to change is small enough that in ca. half of the sequences all 20 beads are coming from the same bag and in ca. half of the sequences the bag of origin changes.” During the instruction, the experimenter emphasized the probabilistic nature of this description and explained that more or fewer bag changes are possible. To support understanding, five practice trials (i.e. five sampled beads) were completed before the main task.

After each bead, participants had 10 seconds to indicate their certainty about the bag of origin. They did so by dragging the marker on a visual analogue scale ranging from 0 = “absolutely sure it comes from the bag with more black beads”, to 1 = “absolutely sure it comes from the bag with more white beads” where 101 different steps on the scale were mapped to probabilities. After each sequence, participants received a visual feedback about the beads they had seen, the bags they had chosen, and the true bag of origin for each bead (see Fig 1). This feedback thus provided a demonstration of how the instructed probabilities could manifest in color changes.

**Fig 2:**
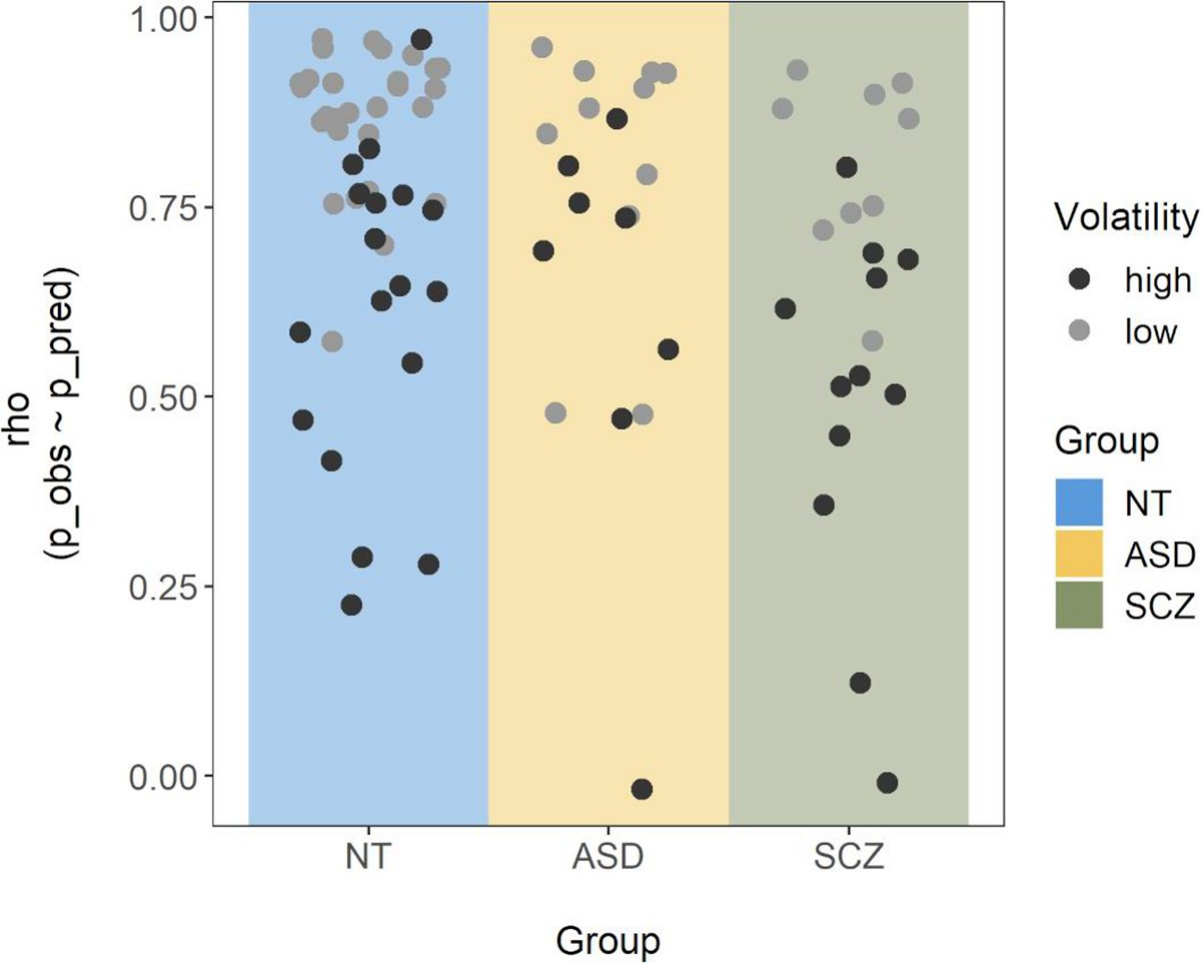
Correlations between observed and predicted probabilities across the task. For each participant, a Spearman correlation was calculated between the participant’s subjective probability ratings and those predicted by the volatility model with the best fitting volatility parameter. Single points represent the corresponding correlation coefficient (*ρ*) for each participant and are colored by size of the corresponding volatility estimate (with clusters of high and low volatility based on a bimodality analysis reported in the results section).

During the task, the instructions, the two bags, and the currently drawn sequence remained on screen. Initial certainty was measured as the average of all indicated probabilities for the first bead of each sequence. Higher values indicate a more JTC-like behavior [14]. Disconfirmatory belief updating was measured as the change in probability rating in favor of a given color whenever this color differed from the color of the two or more preceding beads. The total size of changes was first averaged across the number of occurrences of such events per sequence, and subsequently across sequences. Here, higher values reflect the formerly described over-adjustment behavior [20]. Participants’ perception of the probability for the bags to change, i.e. subjective volatility, was derived from the probabilities participants indicated for each trial *n* out of *N* = 20 in each sequence *k* out of *K* = 5. In an ideal Bayesian model, those probabilities should be based on all observed draws until the current trial *n*, as well as the assumed volatility *v*. A participant’s probability rating ${\tilde{p}}_{k,n}$ should consequently be their guess of the theoretical probability *P*(*x*_*k*,*n*_|*z*_*k*,1_,…,*z*_*k*,*n*_, *v*), where *x* is the bag of origin (*x*_*k*,*n*_ = 0 if bag A, *x*_*k*,*n*_ = 1 if bag B) and *z* is the color of the drawn bead (*z*_*k*,*n*_ = 0 if white, *z*_*k*,*n*_ = 1 if black), with *n* and *k* denoting current number of trial and sequence, respectively, and *v* the probability for a bag change to occur. Volatility *v* was estimated by finding the parameter value that would minimize the difference between the set of theoretical probabilities and the participant’s estimated probabilities ${\tilde{p}}_{k,n}$ (in the least-squares sense). Correlations between observed and predicted probabilities of this ‘volatility model’ indicated model fit and were moderate to high for the majority of participants, but close to zero for three of them (*n*_*ASD*_ = 1, *n*_*SCZ*_ = 2; see Fig 2). Across the sample, model fit correlated negatively with estimated volatility (*ρ* = -.70, *p* < .001), indicating that weaker model fit was associated with higher volatility estimates. For details on the calculation of the theoretical probabilities and parameter estimation, see model description in S1 File. Note that due to the probabilistic nature of the task, the sequences displayed differed between participants. This has the benefit that any observed effects on the group level are independent of the particular sequence chosen. In contrast, administering the same fixed sequence to all participants might introduce particular sequence-dependent biases, which hinders the generalization of any potential results.

#### Visual working memory task

A visual working memory task developed based on previously published paradigms [42, 43] and a variant of the paradigm used by ten Velden Hegelstad and colleagues [44], was administered to measure both visual working memory and implicit metamemory as a proxy for metacognition. An implicit measure was chosen since uncertainty may be encoded without awareness and not accessible to explicit reports [45]. Working memory accuracy was included as a measure to test whether it was related to uncertainty estimation overall and to control for potential differences in cognitive capacity when interpreting group differences on the beads task variables.

**Fig 3:**
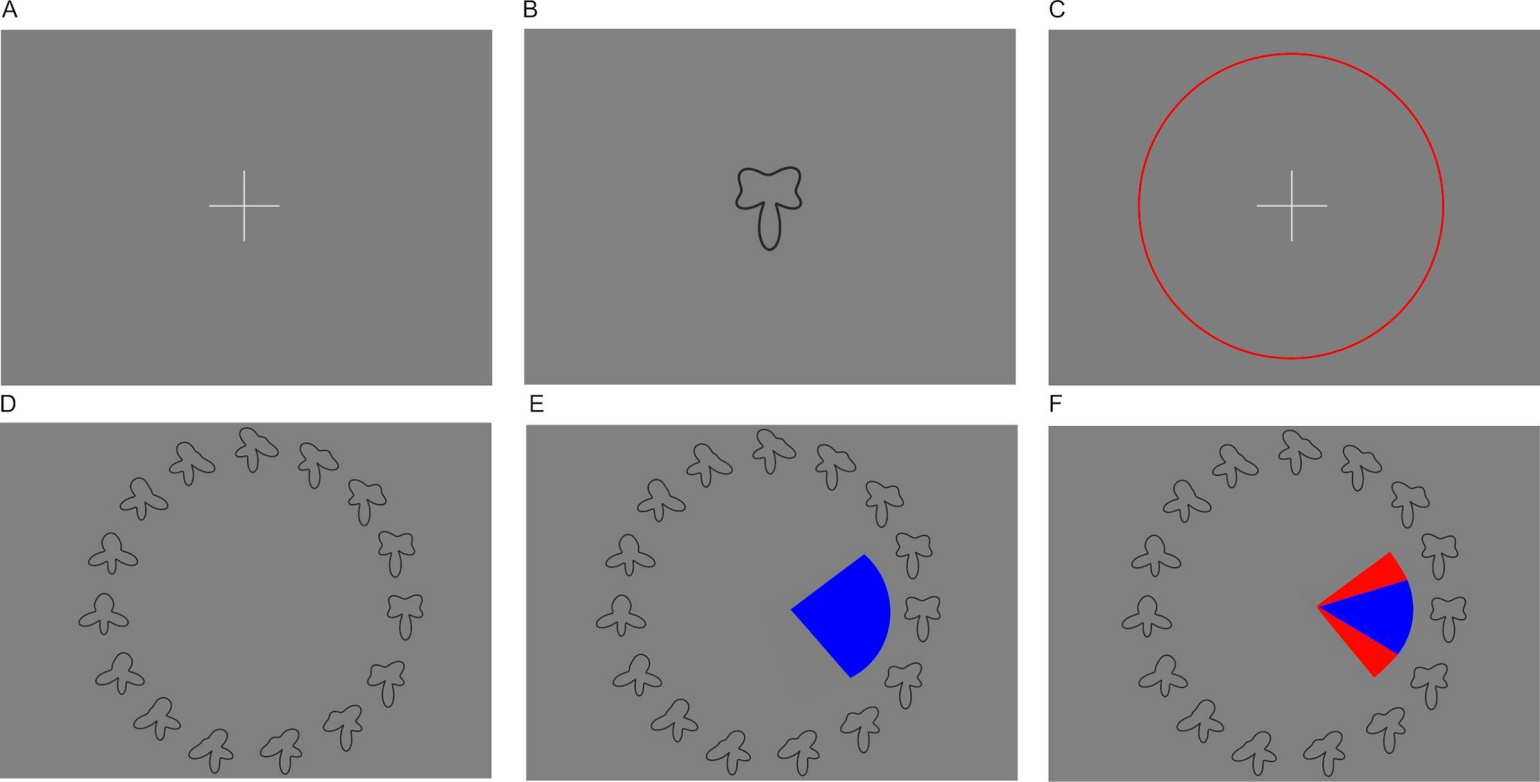
Schematic representation of the visual working memory task. (A) A trial starts with the display of a fixation cross and participants initiate the presentation of the sample shape by clicking on it. (B) The sample shape is then presented for one second, followed by a fixation cross (C). Clicking on it initiates the recall phase (D) in which 30 shapes are presented in a circular arrangement (note that in the example above, only 15 of the 30 shapes, enlarged, are shown for better visibility). (E) The participants now click onto the shape that most resembles what they remember, and set a capture area surrounding it. (F) They receive feedback by being shown the same shape as during the sample phase, correctly placed in the array of shapes. If it is included in the capture area they selected (as in the example above), the excessive part of that capture area is highlighted in red.

A target shape was presented for one second and then had to be selected from an array of similar shapes (see Fig 3). In this array, thirty shapes that varied along continuous quantitative dimensions were displayed in a circular arrangement corresponding to their continuous modification, i.e. shorter angular distance on the circle meant higher resemblance.

The shapes were generated by drawing lines in a polar plot using the following formula:

$$\begin{array}{l} amplitude(phase)\\ \;=10+amplitude2*cos(frequency2*phase+shape)\\ \;+amplitude1*(sin(phase)+1)*sin(frequency1*phase+phase1) \end{array}$$

where "phase" describes the angle relative to the reference direction (upwards) and "amplitude" the length (radius) of the vector. By varying the "shape" parameter in steps of 12 from 0 to 348 degrees, 30 continuously modified shapes were generated (see S1 Fig for a full display of all shapes). The 30 shapes to choose from remained the same across trials and participants, whereas the target shape was selected randomly for each participant on each trial out of the pool of these 30 shapes.

After selection of the target from the array of shapes, participants also set a capture area reflecting their uncertainty about how accurately they had selected the correct shape. They were instructed to set this area big enough to be sure the target shape was included but not bigger than necessary. To demotivate them from capturing the whole circle array at all times, they were rewarded with eight points when their capture area included the target shape, and punished by point subtraction proportional to the size of overshoot when making it too large, though points did not translate to any real reward after task completion. Visual feedback was provided after each trial (see Fig 3). Three practice trials and 30 test trials were administered. Participants had the option to skip trials if they had completely forgotten the sample shape. Trials with extremely large (>350 degrees) or small (<4 degrees) capture areas were excluded from analysis as they might indicate trials where participants accidentally failed to use the option to skip a trial and tried to adjust for that by not setting an appropriate capture area. Visual working memory accuracy was measured as average error, i.e. the average angular distance of the selected shape from the target shape over all trials, with lower values reflecting higher accuracy. Implicit metamemory was assessed by the proportion of all trials where the capture area included the target shape (‘hits’), with lower values indicating overestimation of actual accuracy.

### Procedure

On the day of the assessment, participants were briefed regarding the background of the study and signed the consent form. They then first completed the visual working memory task, followed by the beads task. Duration of each task was ca. 15 minutes, depending on participants’ speed of responding. A short break was introduced between both tasks if required. Task order was not counter-balanced and due to the low similarity and short length of both tasks, no carry-over effects were expected. Demographics were collected on a paper sheet.

### Analysis

One-way ANOVAs were conducted and their residuals tested for normality. Only disconfirmatory belief updating and estimated volatility violated the normality assumption. While disconfirmatory belief updating was log-transformed, estimated volatility followed a bimodal distribution and could not be transformed. Results were therefore verified with Kruskal-Wallis tests and for volatility, exploratory analyses using Gaussian Mixture models were conducted. Significant ANOVA F-Tests were followed by Tukey's Honest Significant Difference tests and effect sizes are reported as η^2^. Significant Kruskal-Wallis tests were followed by Bonferroni corrected Dunn’s Tests and effect sizes are reported as ε^2^. Age and sex did not differ significantly between the groups, and were not controlled for but see analyses in S2 File for group comparisons after propensity matching for both. Level of education differed significantly between the groups but could not be controlled for independently of the diagnosis as the lowest level included more than half of all patients with SCZ but only one participant from the ASD and the NT group. To gauge whether it could be a confounder for any group differences on the task related variables, significant results were followed up by education level comparisons within the NT group. Spearman correlations were chosen to investigate the relationship between the variables of interest across the whole sample. All confirmatory testing was conducted with a significance level of 0.05, one-sided where specified, using the R programming language (R version 3.5.1 [46]).

## Results

Demographic variables are summarized in Table 1.

**Table 1:** Sample demographics per group (total sample size = 86).

		ASD (*n* = 19)			SCZ (*n* = 21)			NT (*n* = 46)		
	*n*	*M* (*SD*)	*Md* (*IQR*)	*n*	*M* (*SD*)	Md (*IQR*)	*n*	*M* (*SD*)	*Md* (*IQR*)	*p*
Sex (m/f)	11/8			17/4			25/21			.11
Education (“1”/”2”/”3”)	1/8/10			12/3/2^a^			1/13/32			< .001
Antipsychotic medication										
Amisulpride				1						
Aripiprazol				5^b^						
Clozapine				2						
Olanzapine				6						
Quetiapine				2						
Risperidone				1						
None				4						
Age		30.32 (8.85)	26 (12)		25.67 (4.74)	26 (7)		28.41 (7.64)	25 (9.75)	.14

Sample sizes (*n*), counts, means (*M*; with standard deviations *SD*) and medians (*Md*; with inter-quartile ranges *IQR*) are displayed. Education was recorded in Norwegian school system categories corresponding to completion of 1 = secondary school (up to age 16), 2 = 6th form college (up to age 19), 3 = higher education (Bachelor, Master, PhD); p-values for group comparisons are provided only for the demographical variables sex and education (Chi-squared tests) as well as Age (ANOVA).

^a^ missing data from 4 patients

^b^ thereof two with additional Quetiapine treatment

**Fig 4:**
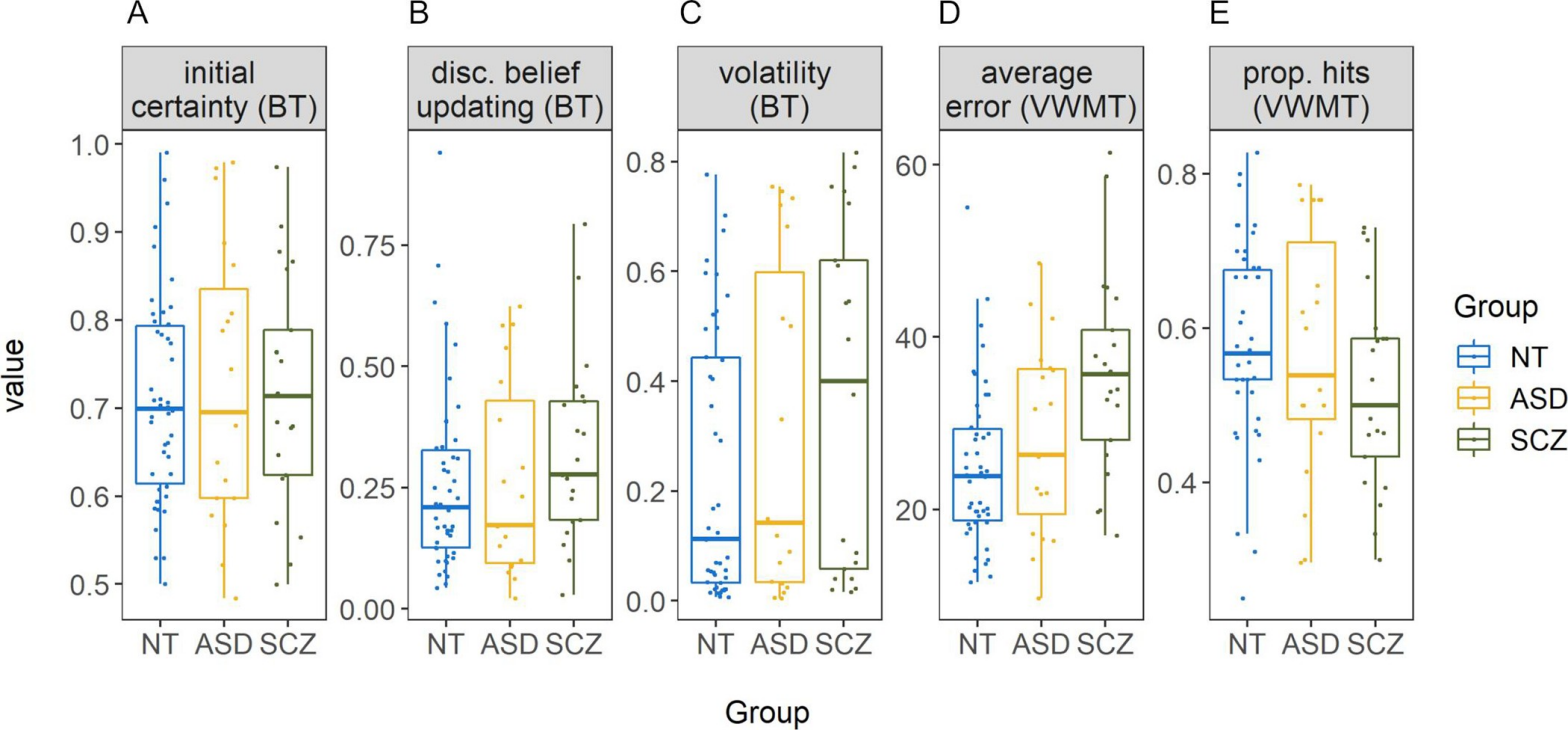
Boxplots per group for all main variables. Beads task (BT) variables are initial certainty (A), untransformed disconfirmatory belief updating (B) and estimated volatility (C). Variables from the visual working memory task (VWMT) include error (D) and proportion of hits (E). NT = neurotypically developing individuals, ASD = individuals with autism spectrum disorder, SCZ = individuals with schizophrenia. All BT variables are expressed as probabilities, average error is expressed in degrees, and proportion of hits is the proportion of trials where the capture area included the target. Points represent single participants.

In the beads task, sequences of beads were drawn randomly for each participant, but group comparisons indicated that on average, all groups experienced approximately the same amount of color changes per sequence, *F*(2,83) = 2.53, η^2^ = 0.06, *p* = .09, with *M*_*ASD*_ = 6.38_,_ *M*_*SCZ*_ = 6.30, and *M*_*NT*_ = 5.79 [nonparametric analysis: χ^2^(2) = 4.88, ε^2^ = 0.06, *p* = .09]. Similarly, the average number of (hidden) bag changes per sequence did not differ by group, *F*(2,83) = 1.19, η^2^ = 0.03, *p* = .31, with *M*_*ASD*_ = 0.65_,_ *M*_*SCZ*_ = 0.75, and *M*_*NT*_ = 0.78 [χ^2^(2) = 2.22, ε^2^ = 0.03, *p* = .33]. Behaviorally, there were no significant group differences in any of the beads task variables: initial certainty, *F*(2,83) = 0.09, η^2^ < 0.01, *p* = .91 [χ^2^(2) = 0.04, ε^2^ < 0.001, *p* = .98] (see Fig 4A); estimated volatility, *F*(2,83) = 1.92, η^2^ = 0.04, *p* = .15 [χ^2^(2) = 3.30, ε^2^ = 0.04, *p* = .19] (see Fig 4C); and log transformed disconfirmatory belief updating, *F*(2,83) = 1.24, η^2^ = 0.03, *p* = .30 [not log transformed for the non-parametric test: χ^2^(2) = 3.16, ε^2^ = 0.04, *p* = .21 (see Fig 4B)]. Average volatility estimates were higher than the instructed value of 0.04 in all groups (see Table 2). Three one-sided one-sample Wilcoxon signed-rank tests confirmed that this was significant for the ASD (*Md* = 0.14, *V* = 167, *p* < .01), the SCZ (*Md* = 0.40, *V* = 216, *p* < .001), and the NT (*Md* = 0.11, *V* = 918, *p* < .001) group. Model fit (correlations between predicted and observed probabilities, see Fig 2) differed significantly between groups, χ^2^(2) = 6.70, ε^2^ = 0.08, *p* = .04, with *Md*_*ASD*_ = 0.79_,_ *Md*_*SCZ*_ = 0.68, and *Md*_*NT*_ = 0.84. Post-hoc comparisons revealed a significant difference between the NT and the SCZ group, *z* = 2.59, *p*_*adj*_ = .03, but not between the ASD and the NT, *z* = -0.78, *p*_*adj*_ > .99, or the ASD and the SCZ group, *z* = 1.48, *p*_*adj*_ = .42. To control for potential learning effects over the course of the task, volatility was additionally estimated separately for the first two and the last two sequences of beads. This revealed a slight decrease in volatility towards the end of the task, possibly related to learning effects in response to the visually provided feedback. However, this volatility change did not differ between groups (see S3 File for details).

**Table 2:** Descriptive summary statistics of the two tasks per group, effect size and p-value from the conducted ANOVAs (total sample size = 86).

	ASD (n = 19)		SCZ (n = 21)		NT (n = 46)		η^2^	*p*
	*M* (*SD*)	*Md* (*IQR*)	*M* (*SD*)	*Md* (*IQR*)	*M* (*SD*)	*Md* (*IQR*)		
Beads task								
initial certainty	0.73 (0.16)	0.70 (0.25)	0.72 (0.13)	0.71 (0.17)	0.71 (0.12)	0.70 (0.18)	<0.01	.90
disconfirmatory belief updating^a^	0.26 (0.20)	0.17 (0.24)	0.33 (0.19)	0.28 (0.18)	0.28 (0.19)	0.21 (0.17)	0.03^b^	.42
estimated volatility	0.30 (0.30)	0.14 (0.56)	0.37 (0.31)	0.40 (0.56)	0.23 (0.24)	0.11 (0.41)	0.04	.16
VWM task								
proportion hits	0.57 (0.16)	0.54 (0.23)	0.52 (0.13)	0.50 (0.15)	0.58 (0.12)	0.57 (0.14)	0.04	.18
error	28.23 (11.07)	26.31 (16.84)	35.72 (11.58)	35.67 (12.77)	24.93 (9.18)	23.88 (10.65)	0.15	< .001

*M* = mean, *SD* = standard deviation, *Md* = median, *IQR* = interquartile range, VWM = visual working memory

^a^ descriptive data not log-transformed but based on original scale

^b^ effect size based on log transformed data

In the visual working memory task, a non-parametric group comparison of number of skipped trials revealed no significant group differences, χ^2^(2) = 1.99, ε^2^ = 0.02, *p* = .37, with *Md*_*ASD*_ = 0.00_,_ *Md*_*SCZ*_ = 0.00, and *Md*_*NT*_ = 0.00. A similar comparison for number of trials where the capture area was out of range (i.e. <4 or >350 degrees), also demonstrated no significant differences between groups, χ^2^(2) = 0.59, ε^2^ = 0.01, *p* = .75, with *Md*_*ASD*_ = 0.00_,_ *Md*_*SCZ*_ = 1.00, and *Md*_*NT*_ = 0.00. There was a significant effect of group on average error (i.e. memory inaccuracy), *F*(2,83) = 8.03, η^2^ = 0.16, *p* < .001 [χ^2^(2) = 12.91, ε^2^ = 0.15, *p* < .01] (see Table 2 and Fig 4D). Post-hoc comparisons revealed that the average error in the SCZ group (*M* = 35.72, *SD* = 11.58) was significantly larger than in the NT group (*M* = 24.93, *SD* = 9.18), *p*_*adj*_ < .001 [nonparametric analysis: *z* = -3.59, *p*_*adj*_ < .001], and numerically but not significantly larger compared to the ASD group (*M* = 28.23, *SD* = 11.07), *p*_*adj*_ = .06 [*z* = -1.97, *p*_*adj*_ = .15]. ASD and the NT group did not differ, *p*_*adj*_ = .47 [*z* = 1.18, *p*_*adj*_ = .71]. Within the NT group, level of education was unrelated to memory inaccuracy, *F*(2,43) = 1.39, η^2^ = 0.06, *p* = .26 [χ^2^(2) = 2.11, ε^2^ = 0.05, *p* = .35]. For proportion of hits (i.e. metamemory), no significant group differences were found, *F*(2,83) = 1.73, η^2^ = 0.04, *p* = .18 [χ^2^(2) = 3.29, ε^2^ = 0.04, *p* = .19] (see Fig 4E). To control for potential effects of response times in the visual working memory task, additional analyses were conducted. These revealed that the SCZ group responded faster on average, but that independent of group membership longer response times were associated with larger errors (see S4 File for details).

**Fig 5:**
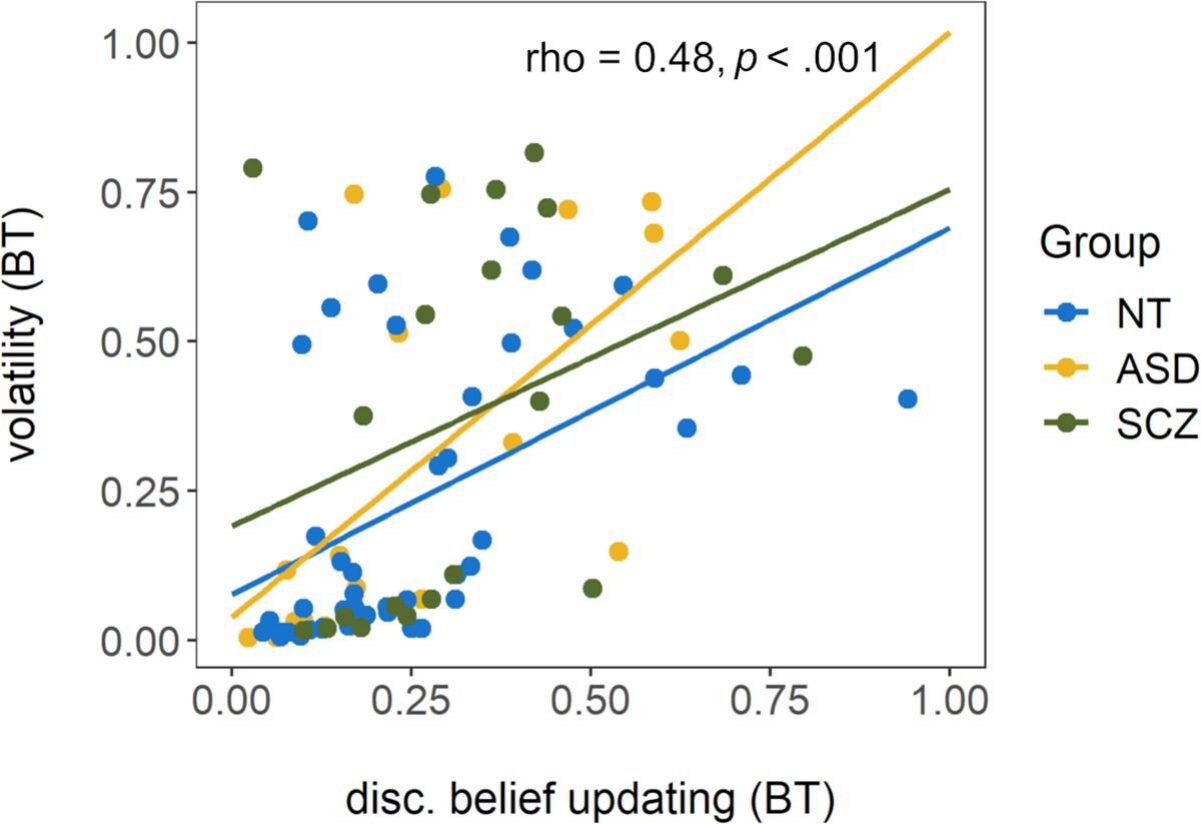
Scatterplot of disconfirmatory belief updating (untransformed) and estimated volatility from the beads task (BT). Rho and *p* display the results of a Spearman correlation conducted across the total sample. Regression lines are fitted for each group for illustrative purposes only. NT = neurotypically developing individuals, ASD = individuals with autism spectrum disorder, SCZ = individuals with schizophrenia.

Disconfirmatory belief updating correlated with both initial certainty (*ρ* = .48, *p* < .001) and estimated volatility (*ρ* = .62, *p* < .001, see Fig 5), but there was no significant relationship between initial certainty and estimated volatility (*ρ* = .09, *p* = .39). There was a strong correlation between proportion of hits (metamemory) and average error (memory inaccuracy) as measured by the visual working memory task (*ρ* = -.59, *p* < .001). Across tasks, average error was positively correlated with disconfirmatory belief updating (*ρ* = .33, *p* < .01) and with estimated volatility (*ρ* = .40, *p* < .001), but not initial certainty (*ρ* = .04, *p* = .68). Proportion of hits was not related to initial certainty (*ρ* = .12, *p* = .28) or disconfirmatory belief updating (*ρ* = -.09, *p* = .42) but was negatively associated with estimated volatility (*ρ* = -.24, *p* = .03). Thus, participants with better metamemory as measured by the visual working memory task also tended to estimate the volatility within the beads task more appropriately, with lower values approaching the true volatility that was introduced by the task design.

As visible in Fig 4C, estimated volatility *v* followed a bimodal distribution, suggesting one high- and one low-volatility cluster. This structure may have masked potential between-group effects in traditional and non-parametric tests. In an exploratory approach, the bimodality of this variable was therefore modeled using Gaussian mixture models in conjunction with a Bayesian estimation method. That approach allowed for the extraction of posterior probability distributions to find the most likely values of the estimated coefficients given the data [47]. The model can be written as

$$p(y|\mu_{1},\mu_{2},\sigma_{1},\sigma_{2},\theta )=\theta Normal(y|\mu_{1},\sigma_{1})+(1-\sigma )Normal(y|\mu_{2},\sigma_{2}),$$

where (*μ*_1_, *σ*_1_) and (*μ*_2_, *σ*_2_) are the parameters of the first and second cluster, respectively, and *θ* is the mixing proportion indicating the relative proportion of subjects who belonged to the first vs. the second cluster. Volatility values (formerly *v*) are labeled as *y* to emphasize the fact that they are treated as data in this estimation context. Prior distributions were specified to be weakly informative [48] with the standard-deviations

σ∼LogNormal(0,0.1),

and the means

μ∼Normal(0,1).

Hamiltonian Monte-Carlo (HMC) methods were applied and implemented in the Stan software [49] using the RStan interface [50]. All models were fitted using four independent chains with 2000 iterations per chain where the first 1000 steps were discarded as warm-up samples. The Gelman-Rubin diagnostic $\hat{R}$ [51] was used to ensure convergence and all $\hat{R}<1.01$. Results are reported in terms of the posterior mean value and the 95% highest-density intervals (HDI) which cover the area in which the true parameter value is located with probability 95% given the model structure. In order to detect group-level effects, parameters *μ*_1_, *μ*_2_ and *θ* were modeled separately per group and the resulting models were compared using leave-one-out cross-validation (LOOCV [52]). Concretely, a sequence of models of increasing complexity was designed and the leave-one-out information criterion (LOOIC) was calculated for each (see Table 3). This criterion can be interpreted similarly as the AIC and BIC criteria (lower values indicate better fit) but is appropriate for Bayesian models.

**Table 3:** Model comparison.

Rank	Free variables between groups	LOOIC	SE(LOOIC)	*Δ*LOOIC	SE(*Δ*LOOIC)
1	*μ*_2_	-70.20	17.05	–	–
2	none	-67.81	17.10	2.39	2.91
3	*μ*_1_ and *μ*_2_	-66.86	16.59	3.34	1.37
4	*μ*_1,_ *μ*_2_ and *θ*	-65.98	16.49	4.22	1.72
5	*μ*_2_ and *θ*	-36.46	13.35	33.74	4.48

**Fig 6:**
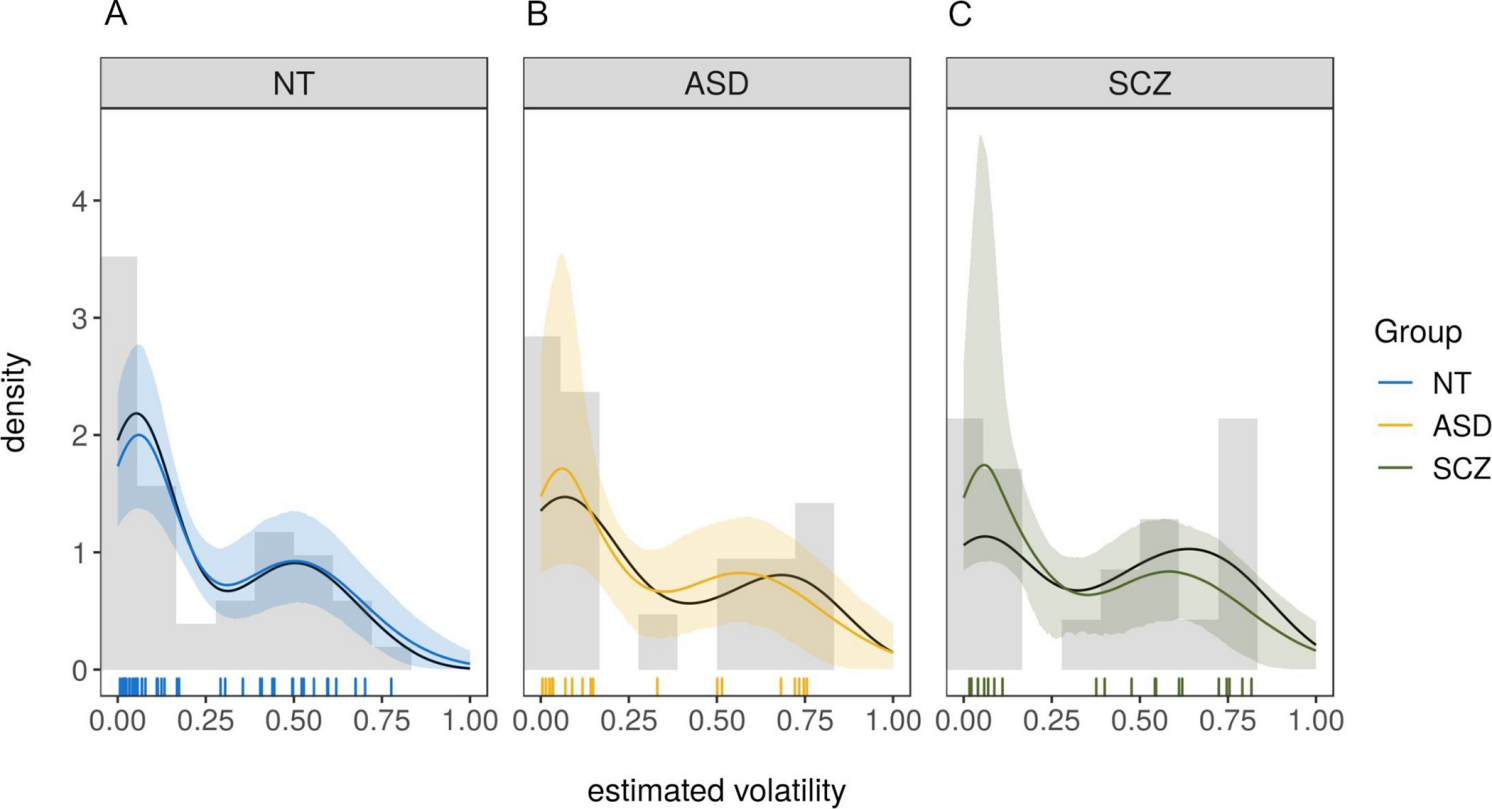
Posterior-predictive distributions of the winning model for all three groups. (A) for neurotypically developing individuals (NT), (B) for individuals with autism spectrum disorder (ASD), (C) for individuals with schizophrenia (SCZ). Colored lines are posterior means of the posterior predictive distributions, shaded areas are the 5% and the 95% percentile. Black lines are the actual data. Vertical lines are the estimated volatility values for each participant based on the Bayesian volatility model as described in section ‘Measures: Beads task’. NT = neurotypically developing individuals, ASD = individuals with autism spectrum disorder, SCZ = individuals with schizophrenia.

As can be seen in Table 3, the model which allowed the mean of the high-volatility cluster (*μ*_2_) to vary between groups performed best in comparison to the baseline-model in which no group-differences were modeled. The model successfully identified two separate clusters, one that was very close to the optimal volatility value of *v*_optimal_ = 0.04 with a cluster mean of *μ*_1_ = 0.05, HDI = [0.04,0.07] and small variance (*σ*_1_ = 0.04, HDI = [0.03,0.06]) and one that was centered at *μ*_2_ = 0.51, HDI = [0.43,0.59] (*σ*_2_ = 0.17, HDI = [0.12,0.22]) reflecting well the bimodal nature of the distribution. Further, the size of the two clusters was very similar with approximately 53% of the subjects belonging to the first (close-to-optimal) cluster, *θ* = 0.53, HDI = [0.42,0.65]. Model-fit was excellent as determined by the posterior predictive distributions for all groups in Fig 6A–6C.

Both the ASD and the SCZ group had a slightly elevated mean in the high-volatility cluster. For the ASD group, the effect was *b*_ASD_ = 0.07, HDI = [-0.05,0.19] with a probability of a truly higher volatility in this cluster compared to the NT group of 89%. For the SCZ group, the effect was *b*_SCZ_ = 0.09, HDI = [-0.01,0.20], with a probability of a truly higher volatility than the NT group of 95%. To check for the effect of education, a median split on estimated volatility was conducted. Within the NT group, level of education was unrelated to volatility ratings being above or below and equal to the median, χ^2^(2) = 0.04, *p* = .98. Further, volatility estimates within the median-split groups did not differ by education (below and equal to median: *F*(1,23) = 2.94, η^2^ = 0.11, *p* = .10 [χ^2^(1) = 2.95, ε^2^ = 0.12, *p* = .09]; above: *F*(2,18) = 0.04, η^2^ < 0.01, *p* = .96 [χ^2^(2) = 0.54, ε^2^ = 0.03, *p* = .76]).

## Discussion

This study investigated probabilistic decision-making and visual (meta-)memory in persons with schizophrenia (SCZ group), persons with high-functioning autism (ASD group) and neurotypically developing individuals without any psychiatric diagnosis (NT group) to explore if and to what extent groups differed in processing of probabilistic information and subsequent estimation of uncertainty. Unexpectedly, none of the groups differed significantly on any of the probabilistic reasoning measures. Relative to NT individuals, neither participants with SCZ nor persons with ASD showed significantly higher or lower certainty when making their first probability rating, when integrating new evidence with previous beliefs or when interpreting the volatility of the task environment. Similarly, none of the groups differed regarding their (un)certainty about their own visual memory performance (metamemory). However, participants with SCZ showed lower visual working memory accuracy than participants of the NT group.

While the absence of a difference between ASD and SCZ group in subjectively perceived volatility is not unexpected in light of the literature that found overestimation of volatility in both groups (e.g. [26, 28]), it is surprising that neither clinical group differed from the NT group. However, additional analyses revealed two clusters of participants: those who estimated volatility in a near-optimal manner and those who strongly overestimated volatility. Within the second cluster, volatility was higher in individuals with ASD and SCZ compared to the NT group, confirming in part the aforementioned findings of volatility overestimation in those clinical groups. The bimodal distribution itself might indicate qualitatively different processing modes [53]. Such processing modes could be related to the different decision-making strategies proposed in the reinforcement learning literature: a model-based mode, which relies on a cognitive representation of state transitions and a complex model of the task overall, and a model-free mode, which is more habitual and driven by trial-and-error feedback [54]. Participants in the low volatility cluster might be more prone to model-based strategies, whereas participants in the high volatility cluster may be more sensitive to trial-wise fluctuations of colors. While both modes are in theory available to all individuals, the choice of one strategy over the other can vary depending on the task at hand and available cognitive resources (e.g. [55]). Notably, volatility was higher for persons with lower working memory accuracy in the current study.

Nevertheless, the absence of overall group differences in the main analyses seems at odds with studies reporting a general overestimation of volatility in individuals with ASD or SCZ. Crucially, most previous studies did not inform their participants about the actual size of the change probability. Instead, it had to be inferred from exposure to the learning environment (e.g. [29, 31]). In contrast, the current study attempted to induce the same prior belief in all groups by providing explicit instructions about the degree of the task environment’s volatility. While one possible explanation of the bimodal volatility distribution is the aforementioned choice of processing mode, another explanation may be individual differences in understanding of the instructions. It is possible that individuals in the high volatility cluster misunderstood the instructions and assumed bags would change with a probability of 0.5 per bead rather than per sequence. Interestingly, miscomprehension of task instructions has been suggested as an explanation for the Jumping-to-Conclusions (JTC) bias in other versions of the beads task [15]. Similarly, misunderstanding of probabilities has been found to explain the JTC bias, possibly caused by reduced general cognitive abilities [56]. In order to clarify the effect of explicit information about volatility on behavior, future studies should contrast conditions where volatility is explicitly announced against conditions where it is not. Further, the role of working memory and other cognitive ability measures in this context should be elaborated, as they may link to the understanding of probabilities and (mis)comprehension of task instructions.

Importantly, while the volatility-estimating model fitted the data of the majority of participants well, model fit was significantly weaker for the SCZ group. Furthermore, weaker model fit was associated with increased volatility estimates across the sample. This may reflect the aforementioned deviation from task instructions or a different choice of processing mode in participants with high volatility values, causing an increased deviation from the behavior the theoretical model would predict. Nevertheless, the estimated volatility values were still those that fitted the observed behavior best, even if not perfectly, and model fit was still reasonable for the majority of participants with high volatility estimates (see Fig 2).

The absence of differences in other, directly observable JTC related variables was surprising. While it was unclear what to expect for participants with ASD given the few and contradictory findings (see [17, 18]), over-adjustment in response to disconfirmatory evidence has been reported for patients with SCZ [20]. This inconsistency with previous findings may in part be related to the choice of method. For SCZ, group differences seem to be less consistent in graded estimates versions of the beads task [16]. Further, the explicit introduction of volatility in the current study may have contributed to the absence of group differences. Similar beliefs about the task’s volatility across groups could cause similar belief updating, as over-adjustment (i.e. increased disconfirmatory belief updating) is likely related to overestimation of volatility: In an environment that is constantly changing, the newest observations seem most reliable and therefore deserve greater attention. This interpretation is supported by the positive correlation between disconfirmatory belief updating and estimated volatility. Hence, introducing volatility explicitly in the current study may have eliminated the difference between persons who typically overestimate volatility (persons with ASD and SCZ) and those that do not (NT group). Importantly, the volatility parameter of the model used in this study is estimated based on all trial-wise deviations of participants’ probabilistic estimates from an ideal Bayesian observer. The model rests on the assumption that these deviations are mainly caused by a misestimation of the true volatility. Yet, other causes for such deviations are conceivable, even if unlikely. As such, estimated volatility might be affected by “noisy” decision-making (see S3 File for additional analyses that address this question). Nonetheless, the positive correlation with disconfirmatory belief updating seems to substantiate the idea that estimated volatility reflects at least in part a belief about the probability for the bags to change, i.e. subjectively perceived volatility of the environment.

The lack of group differences in metamemory could be the result of measuring it implicitly as opposed to former studies that used explicit self-reports (e.g. [36, 37]). It has been suggested that implicit metacognition relies on a different cognitive system than explicit metacognition and is only minimally dependent on working memory [57]. These findings are also in line with recent reports of intact implicit metacognition in SCZ [44] and metacognitive efficiency in first episode psychosis [58]. Interestingly, metamemory was negatively related to estimated volatility. This suggests, that both misestimation of subjective cognitive capacity and overestimation of environmental uncertainty (such as volatility) might be affected by similar mechanisms, potentially driven by higher-level uncertainty calculations in the belief hierarchy of the human mind, and is in line with the conceptualization of aberrant representation of uncertainties as a ‘failure of metacognition’ [24]. Notably, average metamemory scores were rather low, with proportion of hits of 50% to ca. 60% for each group. On the one hand, this might indicate an overall tendency of participants to overestimate the accuracy with which they had remembered and correctly identified the target shape. On the other, this may in part be due to difficulties in perceptually differentiating between the shape stimuli overall, suggesting that the task in that regard might have been slightly too demanding.

The finding of lower visual working memory accuracy only for participants with SCZ relative to the NT group was little surprising. Working memory deficits are well established in SCZ (e.g. [59, 60]) but not in high-functioning autism, where findings are less consistent and performance, particularly in the visual domain, is often unimpaired (e.g. [61, 62]). Lower visual working memory accuracy was related to disconfirmatory belief updating across the whole sample. This fits well with findings that linked the JTC bias to memory performance [40, 59].

Limitations of the current study include the rather small sample sizes for the ASD and the SCZ group. The power of this study might have been too low to detect actual group differences in some of the measures. This is particularly the case for estimated volatility, where descriptive statistics and additional modelling suggest higher values in parts of the SCZ and the ASD group. The study would further have profited from the inclusion of additional cognitive ability measures. It remains unclear to what extent differences in cognitive ability may have attributed to differences in probability estimation and task comprehension. This similarly concerns the findings for visual working memory and JTC, both of which have been linked to general cognitive ability [63, 64]. While possibly related, educational degree was not controlled for in the analyses, as differences in educational levels were so large, that their effects could not be assessed independently of clinical diagnoses. However, within the NT group, education was unrelated to the main variables of interest, though it is noteworthy that the lowest educational level was underrepresented in this group. Groups were not matched by education prior to data analysis as this has been criticized for possibly leading to the selection of an atypical, high-achieving SCZ sample [65].

Further, this comparative approach was purely diagnosis-based and there was no differentiation between patients by symptoms. However, recent studies have not found any correlations between severity of psychopathological symptoms and volatility estimation [32] or aberrant switching behavior [28, 30] in SCZ. For ASD, the relationship is less clear with some studies reporting no relationship between ASD-typical symptoms and volatility-related behavior [66], some suggesting a relationship with few of the behavioral variables [25], and some not investigating any correlations along those lines [12, 27]. It is unclear whether linear relationships should even be expected in a cross-sectional design as some of the symptoms (e.g. delusions in SCZ, rigid behavior in ASD) may constitute a secondary coping mechanism in response to prior volatility overestimation [21, 22]. Regarding the often investigated relationship between JTC like behavior and delusions, results are similarly inconsistent [16, 67], but point towards an absence of this relationship for certainty and responses to contradictory evidence [67]. Furthermore, type or dose of medication were not controlled for in the current study. Antipsychotic medication might worsen or improve cognitive capacity. However, some of the study’s main variables were similar to those investigated in the JTC bias literature and previous findings actually indicated that JTC is not influenced by antipsychotic drugs (e.g. [68, 69]). Finally, the SCZ group was recruited amongst the most severely ill patients (inpatient care) and a majority were males. It is therefore unclear how well the results can be generalized.

To summarize, this study demonstrates reduced visual working memory accuracy of SCZ patients compared to NT controls. Further, the findings did not reveal any group differences for metamemory but suggest higher overestimation of volatility among some participants with autism and schizophrenia. This partially supports the conceptualization of uncertainty misestimation based approaches to phenomenology of these conditions. Nevertheless, despite similarities in social and non-social cognitive performance, both conditions’ symptomatology is heterogeneous in nature and while overlap of some clinical symptoms exists, many of them are rather particular for one of the conditions, respectively (e.g. rigid behavior in ASD, delusions or hallucinations in SCZ). It remains unclear how, if present, similar underlying mechanisms can account for that and future studies should investigate this more closely, linking subjective volatility estimation to clinical symptoms and cognitive ability in a longitudinal design.

## Supporting information

S1 File Mathematical model of the Bayesian observer. (PDF)

S2 File Results of the group comparisons after propensity matching by age and sex. (PDF)

S3 File Additional analyses of beads task variables. (PDF)

S4 File Response time analysis of the visual working memory task. (PDF)

S1 Data (DOCX)

S1 Fig Illustration of all stimuli used in the visual working memory task. Constitutes an exemplary representation of the circle of stimuli in which the target location had to be indicated on each trial. Stimuli are arranged according to their continuous modification. This pool of stimuli and the order of their arrangement were consistent across trials and participants.
(TIF)
