# Supplementary material for: Overestimation of volatility in schizophrenia and autism? A comparative study using a probabilistic reasoning task
Source: PLoS One. 2021 Jan 7;16(1):e0244975. doi: 10.1371/journal.pone.0244975 (PMC7790240; doi:10.1371/journal.pone.0244975)
Supplement: S1 File — (PDF) [file pone.0244975.s001.pdf]

S1 - mathematical model

1 Game rules

- There are two bags, bag A and bag B. The bags are filled with white and black balls. In bag A the fraction of white balls is p, whereas in bag B the fraction of white balls $1-p$.
- The game starts by the administrator drawing one of the bags at random. Let $x_1 = 0$ if the result is bag A and $x_1 = 1$ otherwise. Thus,

$$\mathrm{P}(x_1 = 0) = \mathrm{P}(x_1 = 1) = \frac{1}{2}.$$

- If $x_1 = 0$, the administrator is sampling a ball from bag A at random, and if $x_1 = 1$ the administrator is sampling a ball from bag B at random. The ball sampled is shown to the player and put back into the same bag as it was sampled from. Let $z_1 = 0$ if the draw results in a white ball, and $z_1 = 1$ otherwise. Thus,

$$\mathrm{P}(z_1|x_1) = p^{I(z_1=x_1)}(1-p)^{1-I(z_1=x_1)},$$

  where $I(\cdot)$ equals 1 if the argument is true and zero otherwise.

- For $i = 2, \ldots, n$ sequentially:
  - The administrator puts $x_i = 1 - x_{i-1}$ or $x_i = x_{i-1}$ with probabilities v and $1-v$, respectively.
  - If $x_i = 0$, the administrator is sampling a ball from bag A at random, and if $x_i = 1$ the administrator is sampling a ball from bag B at random. The ball sampled is shown to the player and put back into the same bag as it was sampled from. Let $z_i = 0$ if the draw results in a white ball, and $z_i = 1$ otherwise. Thus,

$$\mathrm{P}(z_i|x_i) = p^{I(z_i=x_i)}(1-p)^{1-I(z_i=x_i)}.$$

- After each ball is shown to the player, the player should
  - say from which bag (s)he thinks the last ball is coming, and
  - give an estimate on the probability that the last ball came from bag A.

2 Wanted results

In this note we discuss how to obtain the following

- Assuming the value of v to be known, compute the ideal Bayesian probability for the last ball to come from bag A, i.e. compute

$$P(x_n|z_1,\ldots,z_n,v) \tag{1}$$

for each value of n.

- Assuming the value of v to be unknown, use the given probability estimates given by the player to estimate the value of v assumed by the player.

3 Computing $P(x_n|z_1,\ldots,z_n,v)$

To find $P(x_n|z_1,\ldots,z_n,v)$, one must first study $P(x_1,\ldots,x_n,z_1,\ldots,z_n|v)$. From the game rules it follows that

$$\begin{aligned}
&\mathrm{P}(x_1,\ldots,x_n,z_1,\ldots,z_n|v) = \mathrm{P}(x_1,\ldots,x_n|v)\cdot\mathrm{P}(z_1,\ldots,z_n|x_1,\ldots,x_n)\\
&= \frac{1}{2}\prod_{i=2}^{n}\left[v^{1-I(x_i=x_{i-1})}(1-v)^{I(x_i=x_{i-1})}\right]\prod_{i=1}^{n}\left[p^{I(z_i=x_i)}(1-p)^{1-I(z_i=x_i)}\right]. \qquad (2)
\end{aligned}$$

We have

$$\begin{aligned}
P(x_n|z_1,\ldots,z_n,v) &= \frac{P(x_n,z_1,\ldots,z_n|v)}{P(z_1,\ldots,z_n|v)}\\
&\propto P(x_n,z_1,\ldots,z_n|v)\\
&= \sum_{x_1}\cdots\sum_{x_{n-1}}P(x_1,\ldots,x_n,z_1,\ldots,z_n|v), \qquad (3)
\end{aligned}$$

where the proportionality is as a function of x_n. To find $P(x_n|z_1,\ldots,z_n,v)$ we therefore need to evaluate the $n-1$ sums in (3) for each possible value of x_n and thereafter scale the result so that the values sum to one. For small values of n direct evaluation of the $n-1$ sums in (3) is computationally feasible, but for larger values of n the Markov structure present in (2) must be utilised to get a computationally efficient procedure. In the following we assume $n \geq 3$. The joint distribution in (2) can then be factorised into

$$\mathrm{P}(x_1,\ldots,x_n|v,z_1,\ldots,z_n) \propto h_{1,2}(x_1,x_2)\cdot h_{2,3}(x_2,x_3)\cdot\ldots\cdot h_{n-1,n}(x_{n-1},x_n), \tag{4}$$

where

$$h_{1,2}(x_1,x_2) = \frac{1}{2}v^{1-I(x_2=x_1)}(1-v)^{I(x_2=x_1)}p^{I(z_1=x_1)}(1-p)^{1-I(z_1=x_1)},$$

$$h_{i-1,i}(x_{i-1},x_i) = v^{1-I(x_i=x_{i-1})}(1-v)^{I(x_i=x_{i-1})}p^{I(z_{i-1}=x_{i-1})}(1-p)^{1-I(z_{i-1}=x_{i-1})}$$

for $i=3,\ldots,n-1$, and

$$h_{n-1,n}(x_{n-1},x_n) = v^{1-I(x_n=x_{n-1})}(1-v)^{I(x_n=x_{n-1})}p^{I(z_{n-1}=x_{n-1})}(1-p)^{1-I(z_{n-1}=x_{n-1})}$$

$$\cdot p^{I(z_n=x_n)}(1-p)^{1-I(z_n=x_n)}.$$

One should note that all the $h_{i-1,i}(x_{i-1},x_i)$ functions also depends on the value of v and the values $z_1,\ldots,z_n$ even if this dependence is not explicitly represented in the notation. Defining

$$g_2(x_2)=\sum_{x_1} h_{1,2}(x_1,x_2) \tag{5}$$

and

$$g_i(x_i)=\sum_{x_{i-1}} g_{i-1}(x_{i-1})h_{i-1,i}(x_{i-1},x_i) \tag{6}$$

for $i=3,\ldots,n$, we get that $g_n(x_n)$ equals the right hand side of (3). Thus,

$$P(x_n|z_1,\ldots,z_n,v)=\frac{g_n(x_n)}{\sum_x g_n(x)}. \tag{7}$$

To evaluate $P(x_n|z_1,\ldots,z_n,v)$ for each possible value of x_n can thereby be done in the following steps.

1. For each $i=2,\ldots,n$, evaluate $h_{i-1,i}(x_{i-1},x_i)$ for each possible combination of values for x_{i-1} and x_i. As the possible values for each of x_{i-1} and x_i is zero and one, four values must be computed for each value of i.

2. Using (5), compute $g_2(x_2)$ for $x_2=0$ and for $x_2=1$.

3. For $i=3,\ldots,n$ in turn, use (6) to compute $g_i(x_i)$ for $x_i=0$ and for $x_i=1$.

4. Using (7), compute $P(x_n|z_1,\ldots,z_n,v)$ for $x_n=0$ and for $x_n=1$.

4 Estimate the value of v used by the player

We now assume the player is using a value of the parameter v when deciding on the probability estimates. We let K denote the number of games or rounds the player is playing, and assume that the player sees N balls in each play. We let $\widetilde{p}_{k,n}$ denote the probability estimate specified by the player after seeing ball number n in play number k. One should note that $\widetilde{p}_{n,k}$ is the players guess on the probability $P(x_{k,n}|z_{k,1},\ldots,z_{k,n},v)$, where $x_{k,n}$ and $z_{k,i}$ corresponds to x_n and z_i, respectively, in Section 3, but where we have now added an index k to distinguish the K rounds played. As the theoretical probability $P(x_{k,n}|z_{k,1},\ldots,z_{k,n},v)$ is a function of v, one can formally estimate the value of v used by the player by finding the value that makes the set of theoretical probabilities $P(x_{k,n}|z_{k,1},\ldots,z_{k,n},v)$ as close as possible to the probability estimates $\widetilde{p}_{k,n}$. More precisely, we suggest to estimate v by minimising the sum of squares of the differences between the probability estimate $\widetilde{p}_{k,n}$ specified by the player and the corresponding theoretical probability $P(x_{k,n}|z_{k,1},\ldots,z_{k,n},v)$. Thus, we define the estimate as

$$\widehat{v}=\underset{v}{\operatorname{argmin}}\left[\sum_{k=1}^{K}\sum_{n=1}^{N}\left(\widetilde{p}_{k,n}-P(x_{k,n}|z_{k,1},\ldots,z_{k,n},v)\right)^2\right]. \tag{8}$$

The minimisation must be done by some numerical minimisation algorithm, within which the theoretical probabilities, $P(x_{k,n}|z_{k,1},\ldots,z_{k,n},v)$, for any value of v can be computed as discussed in Section 3.
