# Supplementary material for: Overestimation of volatility in schizophrenia and autism? A comparative study using a probabilistic reasoning task
Source: PLoS One. 2021 Jan 7;16(1):e0244975. doi: 10.1371/journal.pone.0244975 (PMC7790240; doi:10.1371/journal.pone.0244975)
Supplement: S2 File — (PDF) [file pone.0244975.s002.pdf]

S2 - matched group comparisons

To account for differences in age and sex between the groups, propensity matching was applied, resulting in separately matched subsamples of the neurotypically developing individuals (NT group) for the participants with autism spectrum disorders (ASD group) and the ones with schizophrenia (SCZ group), respectively, as well as a matched subsample of the SCZ group for comparison with the ASD group. After matching, none of the paired samples differed significantly on either age or sex.
To test for group differences, multiple T-Tests were conducted for every single variable of interest. None of the Levene's tests indicated significant heterogeneity of variance for any of the subsample's comparisons. Therefore, no Welch corrections were performed. Effect sizes were calculated as Cohen's *d*. Since estimated volatility and disconfirmatory belief updating were not normally distributed within most of the subgroups, Mann Whitney-U tests were conducted for non-parametric verification of the results. Those results, as well as the associated effect sizes calculated as Cliff's delta, are reported in brackets.

Group comparison for ASD vs. NT:

No significant difference between groups was found for error, $t(36) = 1.28$, $p = .21$, $d = 0.42$; proportion of hits, $t(36) = -0.86$, $p = .39$, $d = -0.28$; initial certainty, $t(36) = -0.44$, $p = .66$, $d = -0.14$; disconfirmatory belief updating, $t(36) = -0.47$, $p = .64$, $d = -0.15$ [U = 153, $p = .44$, Cliff's $d = -0.15$] and estimated volatility, $t(36) = 0.36$, $p = .72$, $d = 0.12$ [U = 186, $p = .89$, Cliff's $d = 0.03$].

Group comparison for SCZ vs. NT:

There was a significant effect of group on average error (i.e. memory inaccuracy) in the VWM task, $t(40) = -2.75$, $p = .01$, $d = -0.85$, indicating a less accurate visual-memory performance in participants with SCZ ($M = 35.72$, $SD = 11.58$) in comparison to healthy controls ($M = 26.34$, $SD = 10.54$).

No significant difference between groups was found for proportion of hits, $t(40) = 0.60$, $p = .55$, $d = 0.19$; initial certainty, $t(40) = 0.61$, $p = .54$, $d = 0.19$; disconfirmatory belief updating, $t(40) = -0.56$, $p = .58$, $d = -0.17$ [U = 180, $p = .32$, Cliff's $d = -0.18$] and estimated volatility, $t(40) = -1.35$, $p = .18$, $d = -0.42$ [U = 171, $p = .22$, Cliff's $d = -0.22$].

Group comparison for ASD vs. SCZ:

The effect of group on average error (i.e. memory inaccuracy) in the VWM task was only marginally significant, $t(36) = -1.94$, $p = .06$, $d = -0.63$, with a (numerically) less accurate visual-memory performance in participants with SCZ ($M = 35.55$, $SD = 12.13$) in comparison to participants with ASD ($M = 28.23$, $SD = 11.07$).

No significant difference between groups was found for proportion of hits, $t(36) = 1.08$, $p = .29$, $d = 0.35$; initial certainty, $t(36) = 0.24$, $p = .81$, $d = 0.08$; disconfirmatory belief updating, $t(36) = -0.85$, $p = .40$, $d = -0.27$ [U = 141, $p = .26$, Cliff's $d = -0.22$] and estimated volatility, $t(36) = -0.83$, $p = .41$, $d = -0.27$ [U = 149, $p = .37$, Cliff's $d = -0.17$].
