# Supplementary material for: Overestimation of volatility in schizophrenia and autism? A comparative study using a probabilistic reasoning task
Source: PLoS One. 2021 Jan 7;16(1):e0244975. doi: 10.1371/journal.pone.0244975 (PMC7790240; doi:10.1371/journal.pone.0244975)
Supplement: S3 File — (PDF) [file pone.0244975.s003.pdf]

S3 – additional analyses of beads task variables

Random or 'noisy' decision-making and volatility

Estimation of subjective volatility via the ideal Bayesian model assumes that deviations between 'ideal' probabilistic responses and the probability ratings made by a participant are largely caused by a misestimation of the true volatility. However, other causes are conceivable. As such, estimated volatility might be affected by "noisy" or "random" decision-making. Notably, it is difficult to conceptually differentiate such "noise" from volatility, as volatility per se might be the cause driving "noise" or seemingly "random" choice behavior.

Nevertheless, to obtain an approximate estimate of "random" or "noisy" behavior in the beads task, an additional measure was constructed based on all those occurrences where when a bead was of the same color as the previous two, the belief was updated into the *opposite* direction, i.e. the belief in the currently presented colors was decreased.

Example: a participant sees three white beads in a row and indicates a probability for them to originate from the bag with more white beads as 0.7 and 0.8 for the first two trials. On the third trial, they then *decrease* their belief to 0.7 again when actually, given the evidence, they should keep *increasing* their belief certainty about the beads to originate from the bag with more white beads.

Such "random belief updating" was calculated as the mean change in belief across all occurrences of this kind for each sequence, averaged over number of sequences for each participant.

A non-parametric Kruskal-Wallis test (due to the high positive skewness in random belief updating) revealed no significant group difference, $\chi^2(2) = 3.32$, $p = 0.19$, $\varepsilon^2 = 0.04$.

Across groups, random belief updating was strongly and positively associated with volatility, $\rho = .63$, $p < .001$. While this might suggest that estimated volatility largely reflected noise or random behavior, it is important to consider that a conceptual distinction between both concepts may not fully be valid. After all, "random" belief changes may indeed be caused by an increased belief about the frequency with which the bag of origin is secretly changed (volatility), even in the absence of obvious evidence for an occurred change.

Importantly, volatility was also strongly related to disconfirmatory belief updating. Here, the conceptual relationship between both variables is slightly more obvious: in an unstable environment, disconfirmatory evidence might suggest an occurred change – so the larger one thinks the probability is for a change to occur, the more one will react to disconfirmatory evidence in terms of belief updating.

An additional analysis was conducted to gauge to what extent both random and disconfirmatory belief updating contributed to estimated volatility. Participants were divided into groups with high (above the median) or low (below or equal to the median) volatility estimates. A logistic regression was conducted on volatility group membership (0 = low, 1 = high), including main

effects of both random and disconfirmatory belief updating, both standardized. McFadden's R^2 of this model was .40, and the Odds Ratio was 10.12 for (standardized) random belief updating [CI 2.5%: 2.64, 97.5%: 53.23] and 1.92 for (standardized) disconfirmatory belief updating [CI 2.5%: 1.92, 97.5%: 10.62]. This demonstrates that even if random belief updating was interpreted as a pure measure of ”noise” caused by different factors than an overestimation of volatility, when accounting for its contribution to volatility there remains a significant contribution of disconfirmatory belief updating, a variable which is clearly also conceptually related to volatility.

Volatility change throughout the task

Since feedback was provided after every completed sequence in the beads task, learning processes may have caused a decrease in subjective volatility over time. In the original volatility model, subjective volatility was estimated based on all sequences. To explore whether volatility estimates might have decreased over time, the model was refitted to the first two and the last two sequences, respectively. Volatility change was then calculated by subtracting volatility estimated for the first two sequences from volatility estimated for the last two sequences for each participant, with values below zero indicating a decrease of volatility towards the end of the task.

A one-sided one-sample Wilcoxon signed-rank test (due to the non-normality of the volatility change variable) on data of the whole sample confirmed that indeed, this change was significantly below zero across participants, $Md = -0.01$, $V = 1260$, $p < .01$.

To assess whether groups differed in terms of this volatility change, a Kruskal-Wallis test was applied. This did not reveal any significant group differences, $\chi^2(2) = 0.77$, $\varepsilon^2 = 0.06$, $p = .68$, indicating that groups learned similarly from feedback.
