# Supplementary material for: Overestimation of volatility in schizophrenia and autism? A comparative study using a probabilistic reasoning task
Source: PLoS One. 2021 Jan 7;16(1):e0244975. doi: 10.1371/journal.pone.0244975 (PMC7790240; doi:10.1371/journal.pone.0244975)
Supplement: S4 File — (PDF) [file pone.0244975.s004.pdf]

S4 - response time analysis (VWMT)

Response times in the visual working memory task (VWMT) were compared between groups. They were recorded as the time between a) appearance of the array from which the target shape had to be selected and b) finalization of the capture area. For each participant, invalid trials (i.e. skipped trials or trials where the capture area failed to meet the inclusion criteria) as well as response times larger than mean response time + 2.5*standard deviation or smaller than mean response time - 2.5*standard deviation were excluded. An ANOVA conducted on the log-transformed average response times showed a significant group effect, $F(2,83) = 7.99$, $\eta^2 = 0.16$, $p < .001$, with $M_{ASD} = 2.64$, $M_{SCZ} = 2.24$, and $M_{NT} = 2.47$ (not log-transformed means: $M_{ASD} = 14.76$, $M_{SCZ} = 9.87$, and $M_{NT} = 12.55$). Post-hoc comparisons revealed that response times in the SCZ group were significantly shorter as compared to both the NT, $p_{adj} = .02$, and the ASD group, $p_{adj} < .001$. Response times did not differ significantly between the ASD and the NT group, $p_{adj} = .15$.

Notably, shorter response times may on the one hand indicate that a reduced amount of time is allocated to the processing of the visually presented information. Accordingly, shorter response times in the SCZ group might explain the decreased recall performance. On the other hand, shorter response times may reflect increased certainty about the upcoming choice (see e.g. Rahnev et al., 2020) and as such might generally predict increased performance. To elucidate these questions, a linear mixed-effects model was specified, with trial-wise recall error (i.e. deviation from target) as outcome, and trial-wise log transformed response times, group, and their interaction as predictors, including a random intercept for participant number. This revealed a significant positive association between response times and error, meaning recall accuracy was lower on trials where response times were longer (see Table S5). However, this relationship did not differ significantly between NT and SCZ, or NT and ASD group. Taken together, these results suggest that the reduced memory accuracy observed in the SCZ group cannot be explained by their tendency to respond faster.

Table S5. Linear mixed-effects model results for error in the visual working memory task.

	b	*t*	*p*
log(RT)	6.95	3.06	< .01
ASD	-1.78	-0.17	.87
SCZ	21.47	2.39	.02
log(RT)*ASD	1.77	0.44	.66
log(RT)*SCZ	-4.00	-1.05	.30

Notes: log(RT) = log-transformed response time; baseline for group effects: neurotypical control group

References (suppl.)

Rahnev, D., Desender, K., Lee, A. L., Adler, W. T., Aguilar-Lleyda, D., Akdoğan, B., ... & Bègue, I. (2020). The confidence database. *Nature human behaviour*, *4*(3), 317-325.
