# Supplementary material for: Overestimation of volatility in schizophrenia and autism? A comparative study using a probabilistic reasoning task
Source: PLoS One. 2021 Jan 7;16(1):e0244975. doi: 10.1371/journal.pone.0244975 (PMC7790240; doi:10.1371/journal.pone.0244975)
Supplement: S1 Data — (DOCX) [file pone.0244975.s005.docx]

# Additional information

Anonymized raw and processed data as well as the supporting information (S1 – S5) are available in an Open Science Framework repository: DOI 10.17605/OSF.IO/UCA5E.
