# Supplementary figures and images for: Overestimation of volatility in schizophrenia and autism? A comparative study using a probabilistic reasoning task
Source: PLoS One. 2021 Jan 7;16(1):e0244975. doi: 10.1371/journal.pone.0244975 (PMC7790240; doi:10.1371/journal.pone.0244975)

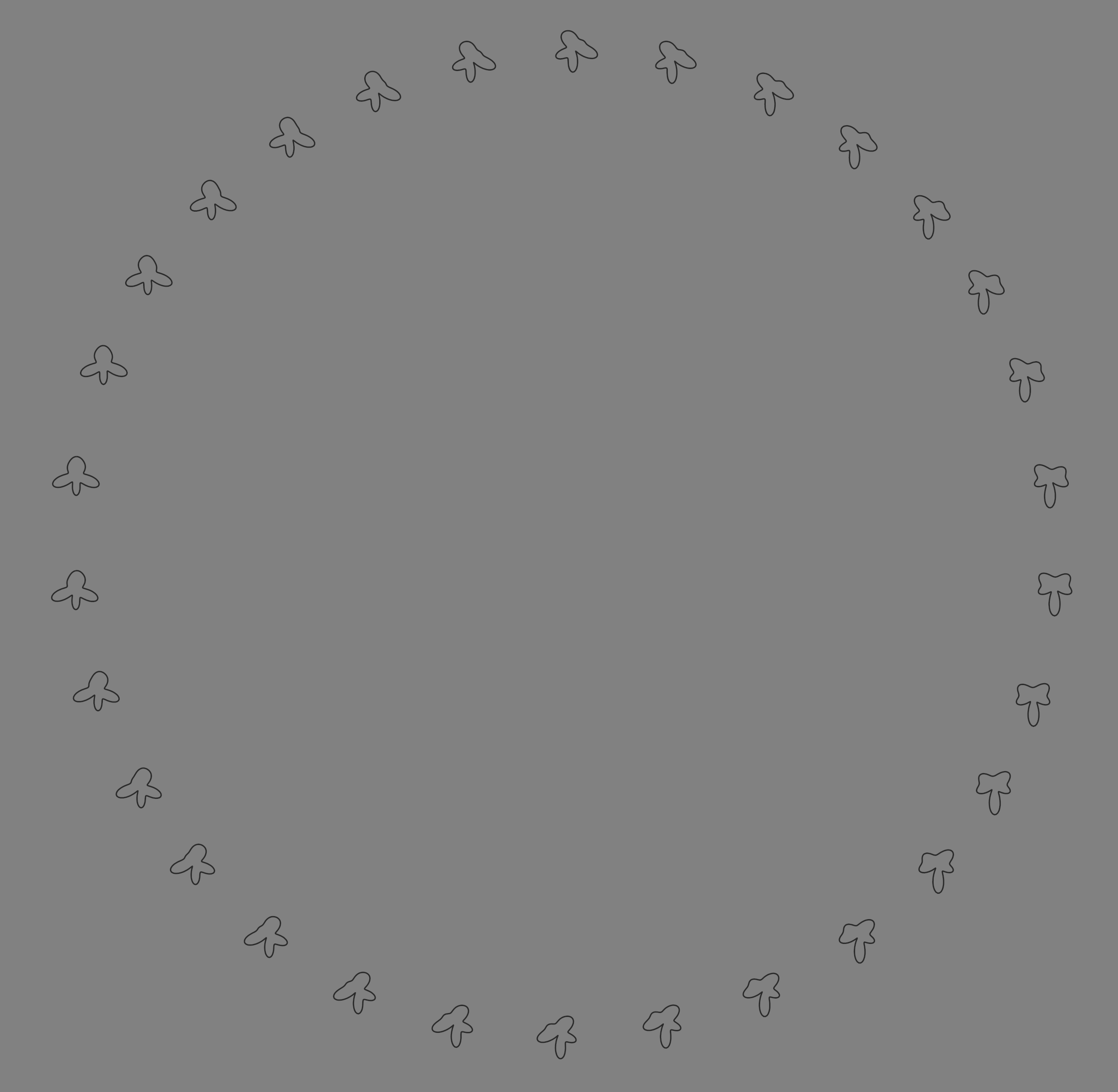

Supplement: S1 Fig — Constitutes an exemplary representation of the circle of stimuli in which the target location had to be indicated on each trial. Stimuli are arranged according to their continuous modification. This pool of stimuli and the order of their arrangement were consistent across trials and participants. (TIF) [file pone.0244975.s006.tif]
